# Identification and characterization of differentially expressed circular RNAs in extraocular muscle of oculomotor nerve palsy

**DOI:** 10.1186/s12864-023-09733-3

**Published:** 2023-10-17

**Authors:** Mingsu Shi, Yanxi Fang, Yu Liang, Yuxiang Hu, Jiaqiu Huang, Weiyi Xia, Hewei Bian, Qiao Zhuo, Lianqun Wu, Chen Zhao

**Affiliations:** 1 Eye Institute, Department of Ophthalmology, Eye & ENT Hospital, Fudan University, 83 Fenyang Road, Shanghai, 200031 China; 2 NHC Key Laboratory of Myopia (Fudan University), Key Laboratory of Myopia, Chinese Academy of Medical Sciences, 83 Fenyang Road, Shanghai, 200031 China; 3 Shanghai Key Laboratory of Visual Impairment and Restoration, 83 Fenyang Road, Shanghai, 200031 China

**Keywords:** Circular RNA, Extraocular muscle, Oculomotor nerve palsy, Constant exotropia

## Abstract

**Background:**

Oculomotor nerve palsy (ONP) is a neuroparalytic disorder resulting in dysfunction of innervating extraocular muscles (EOMs), of which the pathological characteristics remain underexplored.

**Methods:**

In this study, medial rectus muscle tissue samples from four ONP patients and four constant exotropia (CXT) patients were collected for RNA sequencing. Differentially expressed circular RNAs (circRNAs) were identified and included in functional enrichment analysis, followed by interaction analysis with microRNAs and mRNAs as well as RNA binding proteins. Furthermore, RT-qPCR was used to validate the expression level of the differentially expressed circRNAs.

**Results:**

A total of 84 differentially expressed circRNAs were identified from 10,504 predicted circRNAs. Functional enrichment analysis indicated that the differentially expressed circRNAs significantly correlated with skeletal muscle contraction. In addition, interaction analyses showed that up-regulated circRNA_03628 was significantly interacted with RNA binding protein AGO2 and EIF4A3 as well as microRNA hsa-miR-188-5p and hsa-miR-4529-5p. The up-regulation of circRNA_03628 was validated by RT-qPCR, followed by further elaboration of the expression, location and clinical significance of circRNA_03628 in EOMs of ONP.

**Conclusions:**

Our study may shed light on the role of differentially expressed circRNAs, especially circRNA_03628, in the pathological changes of EOMs in ONP.

**Supplementary Information:**

The online version contains supplementary material available at 10.1186/s12864-023-09733-3.

## Introduction

Strabismus is divided into comitant strabismus and incomitant strabismus. Comitant strabismus is described as having an equal angle of ocular misalignment in all fields of gaze, as well as a full range of eye movements no matter which eye is used for fixation [1]. Conversely, incomitant strabismus occurs when the eye movement is restricted due to paralytic or mechanical causes [1]. Strabismus caused by oculomotor nerve palsy (ONP) is a kind of incomitant strabismus, featuring a complete or partial ophthalmoplegia innervated by the oculomotor nerve, including levator palpebrae superioris, inferior rectus, superior rectus, medial rectus, inferior oblique and pupillary sphincter [2].

The etiologies of ONP vary from vascular related causes such as aneurysm, which is the leading cause, to idiopathic causes, trauma, and neoplasm [3, 4]. Patients with ONP typically present with diplopia, ptosis, and less commonly, awareness of an enlarging pupil [5]. Treatments may require neurosurgical intervention as well as strabismus surgery, depending on the visual acuity of the patients, the cause of their ONP, the angle of deviation, and the presence of amblyopia [6]. Although appropriate surgical procedures can be rewarding for ONP patients [7–9], the long-term outcomes between different surgical options showed no statistically significant difference [10]. Therefore, further insight into ONP is urgently needed for better evaluation and management of ONP.

RNA molecules with a single strand and a covalently closed structure are known as circular RNAs (circRNAs), which are ubiquitous in all life forms including viruses and mammals [11]. In recent studies, circRNAs have been discovered to function in multiple ways, including being translated into polypeptides and acting as protein scaffolds or microRNA (miRNA) sponges [12–14]. The regulatory role of circRNAs has been revealed in several ophthalmologic diseases, such as circRNA ZNF532 in diabetes-induced retinal vascular dysfunction and pericyte degeneration [15], and circRNA ZBTB44 in the development of choroidal neovascularization [16]. In addition, circRNAs have been proven to have an implication on the myogenesis and atrophy of skeletal muscles [17, 18], which is involved in skeletal muscle diseases such as Duchenne muscular dystrophy [19]. However, the role of circRNAs in ONP remains unknown.

Therefore, we tried to elaborate the role of circRNAs in the extraocular muscles (EOMs) of ONP in this study. High-throughput RNA sequencing was conducted on medial rectus muscle tissue samples from ONP and constant exotropia (CXT) patients. Differentially expressed circRNAs were identified and included in functional enrichment analysis. Afterwards, interaction networks between differentially expressed circRNAs and miRNAs and mRNAs as well as RNA binding proteins (RBPs) were constructed to further analyze the possible role of circRNAs in EOMs of ONP. Furthermore, we validated the differential expression of several circRNAs by real-time quantitative polymerase chain reaction (RT-qPCR). Finally, we elaborated and verified the localization and expression of the up-regulated circRNA_03628, whose clinical significance was examined by receiver operating characteristic (ROC) curve analysis. Our study may provide a circRNA expression profile in EOMs of ONP, and reveal the possible role of circRNAs, especially circRNA_03628, in the pathological changes and clinical evaluation of ONP.

## Methods

### Subjects and tissue samples

Our study enrolled two groups of patients diagnosed with ONP (congenital or acquired) and CXT (infantile exotropia or intermittent exotropia presenting as a constant pattern) from September 2019 to February 2022 in the Eye and ENT (EENT) Hospital, Fudan University, China. A single surgeon (C.Z.) operated on all patients for surgical procedures of medial rectus strengthening and lateral rectus recession. Patients with previous strabismus surgery, any other cranial nerve palsy, any other disease involving the EOMs, or follow-up time < 3 months were excluded.

Before surgery, each patient underwent a complete ocular examination as well as sensory and motor evaluation as previously published [20]. In brief, a prism alternating cover test (PACT) was used to measure the horizontal and vertical deviations. For the paretic eyes and patients who could not participate in PACT, a Krimsky test was applied. Duction deficits were measured on a − 5 to 0 scale, with − 5 indicating the inability of the eye to achieve midline; −4, the ability to just reach midline; −3, the ability to cross midline but with 75% deficit left; −2, the ability to cross midline but with 50% deficit left; −1, the ability to cross midline but with 25% deficit left [21, 22].

Medial rectus muscle samples of both the ONP and CXT groups were obtained from the wastes of strabismus surgery, and immediately stored in a -80 °C freezer.

### RNA extraction and sequencing analysis

Total RNA was isolated from the medial rectus muscle samples taken from four ONP patients and four CXT patients and stored in TRIzol reagent (Invitrogen, Carlsbad, CA, USA) following the manufacturer’s instructions. The quality and quantity of the total RNA samples was measured using Nanodrop 2000 (Thermo Scientific, Wilmington, DE, USA). Samples with an OD260/280 ratio between 1.8 and 2.1 were accepted. Afterwards, the integrity of the RNA samples was determined using an Agilent 2100 Bioanalyzer (Agilent Technologies, Santa Clara, CA, USA). Samples with an RNA Integrity Number (RIN) ≥ 8 were utilized for further analysis.

RNA sequencing libraries were established using a ribosomal RNA-depleted RNA by TruSeq Stranded Total RNA Library Prep Kit (Illumina, San Diego, CA USA). The Agilent 2100 Bioanalyzer (Agilent Technologies) was utilized for library quality control. Raw reads of fastq format were firstly processed using fastp [23] and the low-quality reads were removed to obtain the clean reads. Quality control details for each sample were displayed in Supplementary Table 1. High-throughput RNA sequencing of total RNA and small RNA was conducted on an Illumina NovaSeq 6000 System (Illumina) with a paired-end run performed with a 150 bp read length [24].

### CircRNA prediction, annotation and quantification

Sequence Alignment/Maps (SAM) files were generated by aligning the sequencing reads of each medial rectus muscle sample with a reference genome from Genome Database (version GRCh38.p12) using the BWA-MEM algorithm. Afterwards, the paired chiastic clipping signals were scanned by CircRNA identifier (CIRI) software [25]. Junction reads as well as GT-AG splicing signals were used to predict circRNA sequences [26].

Predicted circRNA transcripts were annotated according to the length and exon numbers of circRNAs and their distribution on the chromosome. In addition, BEDTools software [27] was used to classify the positional relationship of circRNAs to known protein-coding transcripts. Meanwhile, a comparison between the predicted circRNAs and those recorded in circAtlas (https://ngdc.cncb.ac.cn/circatlas/) [28], circBase (http://www.circbase.org/) [29] and CIRCpedia v2 (http://yang-laboratory.com/circpedia/) [30] databases was carried out.

CircRNA expression levels were quantified using the reads per million (RPM) algorithms, which can be calculated using the following formula:

$$RPM=\frac{number of circular reads}{number of total reads \left(units in million\right)}$$

where, “number of circular reads” indicates the number of reads aligned to the back-spliced junction region of circRNAs, while “number of total reads” indicates the number of clean reads obtained from the sequencing data of each sample.

### Small RNA library construction and sequencing analysis

Small RNA libraries were built utilizing TruSeq Small RNA Sample Prep Kits (Illumina) from each sample in accordance with the manufacturer’s instructions. In brief, total RNA was ligated to adapters at each end, reverse transcribed to cDNA and PCR amplified. PCR products ranging between 140 and 160 bp were isolated and used to create small RNA libraries. DNA High Sensitivity Chips was used to analyze the quality of the libraries on the Agilent Bioanalyzer 2100 system (Agilent Technologies). Low quality reads were filtered, and the reads with 5’ primer contaminants and polyA were removed. The reads without a 3’ adapter or an insert tag, shorter than 15 nt, or longer than 41 nt were filtered, and the clean reads were obtained. Quality control details for each sample were displayed in Supplementary Table 1. Finally, the Illumina NovaSeq 6000 System (Illumina) was used to sequence the libraries, generating 150 bp paired-end reads.

### MiRNA prediction, annotation and quantification

RawData was obtained through base calling analysis of the original sequencing data. We excluded primers and adapter sequences from RawData. Quality control and length screening were then conducted. Clean reads were compared with the reference transcripts and aligned to cDNA sequences, Rfam database v10.1 (http://www.sanger.ac.uk/software/Rfam) [31] and Repbase database [32] using Bowtie software [33] to exclude irrelevant sequences. Afterwards, the sequences were aligned to miRNAs from miRBase (version 22.0) [34] to annotate known miRNAs. The unannotated sequences were collected for new miRNA prediction.

Based on the transcript per million (TPM) algorithm [35], expression levels of known and new predicted miRNAs were quantified using the following formula:

$$TPM=\frac{N}{M}*{10}^{6}$$

where, “N” indicates the number of reads aligned for each miRNA, and “M” indicates the number of clean reads in the sequencing data of each sample.

### Differential expression analysis and functional enrichment analysis

DESeq package [36] in R was utilized to standardize the count data of circRNAs and miRNAs in each sample. Fold change (FC) was calculated and the significance of difference was tested by the negative binomial (NB) distribution test based on the count data. CircRNAs and miRNAs with differential expression were screened using the cut-off criteria of *P* value < 0.05 and |log2 FC| > 1.

Gene Ontology (GO) functional enrichment analysis (http://geneontology.org/) of the parent genes of the differentially expressed circRNAs was conducted utilizing category of biological process (BP), with the cut-off criterion of *P* value < 0.05. In addition, Kyoto Encyclopedia of Genes and Genomes (KEGG) database (https://www.kegg.jp/) was utilized to show the pathway enrichment of the parent genes of differentially expressed circRNAs [37], with the cut-off criterion of *P* value < 0.05.

### CircRNA-miRNA-mRNA network building

CircRNAs contain miRNA binding motifs and can function as miRNA sponges [13]. In order to estimate the possible function of circRNAs in EOMs of ONP, Miranda software [38] was utilized to predict the interactions between potential circRNA-miRNA and miRNA-mRNA pairs with the threshold of *P* value < 0.05. According to the predicted miRNA binding motifs, the circRNA-miRNA-mRNA network was formed for circRNAs which are differentially expressed in each sample of the ONP group. The diagram was drawn using an R network package (https://CRAN.R-project.org/package=network).

### CircRNA-RBP interaction analysis

RBPsuite [39] was utilized for the prediction of RBPs and the calculation of RBP binding scores with up-regulated circRNAs (< 4000 nt). CircRNA-RBP binding sites were predicted using CircRNAs Interact with Proteins (CRIP) [40]. 101 nucleotides of the input circRNA were broken into segments. The interaction between the segments and the RBPs was assessed and scored. Subsequently, the RBPsuite detected the verified motifs on the binding segments and gave a distribution of the binding scores across the entire sequence.

### Quantification of circRNAs utilizing RT-qPCR

In order to further validate the RNA sequencing results, RT-qPCR was used to detect circRNA expression. First, we isolated total RNA from the medial rectus muscle samples from 16 ONP patients and 10 CXT patients using TRIzol reagent (Invitrogen). The cDNA was then reverse-transcribed from 500ng of extracted total RNA with Takara Bio Company’s PrimeScript RT Reagent Kit (Takara Bio Company, Otsu, Shiga, Japan) and amplified with a SYBR Green Kit (Takara Bio Company) on a LightCycler® 480 II Real-time PCR Instrument (Roche, Basel, Switzerland). After normalizing the circRNAs to housekeeping gene GAPDH, the 2^−ΔΔCt^ method was used to quantify gene expression levels. All of the primers were synthesized by Generay Biotech (Generay, Shanghai, China). FastPCR [41] was used to calculate the primer efficiency of each primer, all with an efficiency over 85%. The primer sequences are listed in Table 1.

**Table 1:** Primer sequences for RT-qPCR analysis

Name	Primer Sequence (5’-3’)
circRNA_03628	F: AGTCTACAGTCCCGAATTCTAT R: TATTGGAGACATGCAGCCG
circRNA_04725	F: CCAGAGACCAACGAGATCC R: TGTAGCTCCCGAAGCAATG
circRNA_04985	F: TCAAGCCTAGAGGCAACC R: CGGGCTGTACCAGATGTAT
circRNA_06396	F: GGCATCATGAGGCAGAGATTA R: GAGTCTCTGGGTGACCCTTA
circRNA_03621	F: GCCGTCTACTCCCATGTCA R: TCGACAGAAACACATCCAGGA
circRNA_02140	F: AGGCAAATATGAGCAGGGGT R: AGCGTGAAAGAAATGGCAGG
circRNA_02453	F: TGTCCGACCCTACGAAAAGG R: GGATCGCTTTGAAGAGCAGC
GAPDH	F: GGAGCGAGATCCCTCCAAAAT R: GGCTGTTGTCATACTTCTCATGG

### Further elaboration of the up-regulated circRNA_03628

The location of the parent gene of circRNA_03628 on genome was obtained from National Center for Biotechnology Information (NCBI). Then LncLocator 2.0 was utilized to predict the localization of circRNA_03628 at the subcellular level (http://www.csbio.sjtu.edu.cn/bioinf/lncLocator2/). LncLocator is an ensemble predictor, combining four learning machines using a stacked ensemble strategy. It trains an end-to-end depth model of each cell line for predicting circRNA subcellular localisation from sequences. The source code can be found at https://github.com/Yang-J-LIN/lncLocator2.

Afterwards, Sanger sequencing and nucleic acid electrophoresis were carried out to verify the back-splice junction of circRNA_03628. The expression of circRNA_03628 in multiple tissues was obtained from circAtlas 2.0 (http://159.226.67.237:8080/new/index.php). Furthermore, ROC curve analysis was utilized to further elucidate the clinical importance of circRNA_03628. The area under the curve (AUC) was calculated based on the ROC curve. A higher AUC indicates better sensitivity and specificity (maximum AUC = 1).

## Results

### CircRNA identification and annotation

**Fig. 1:**
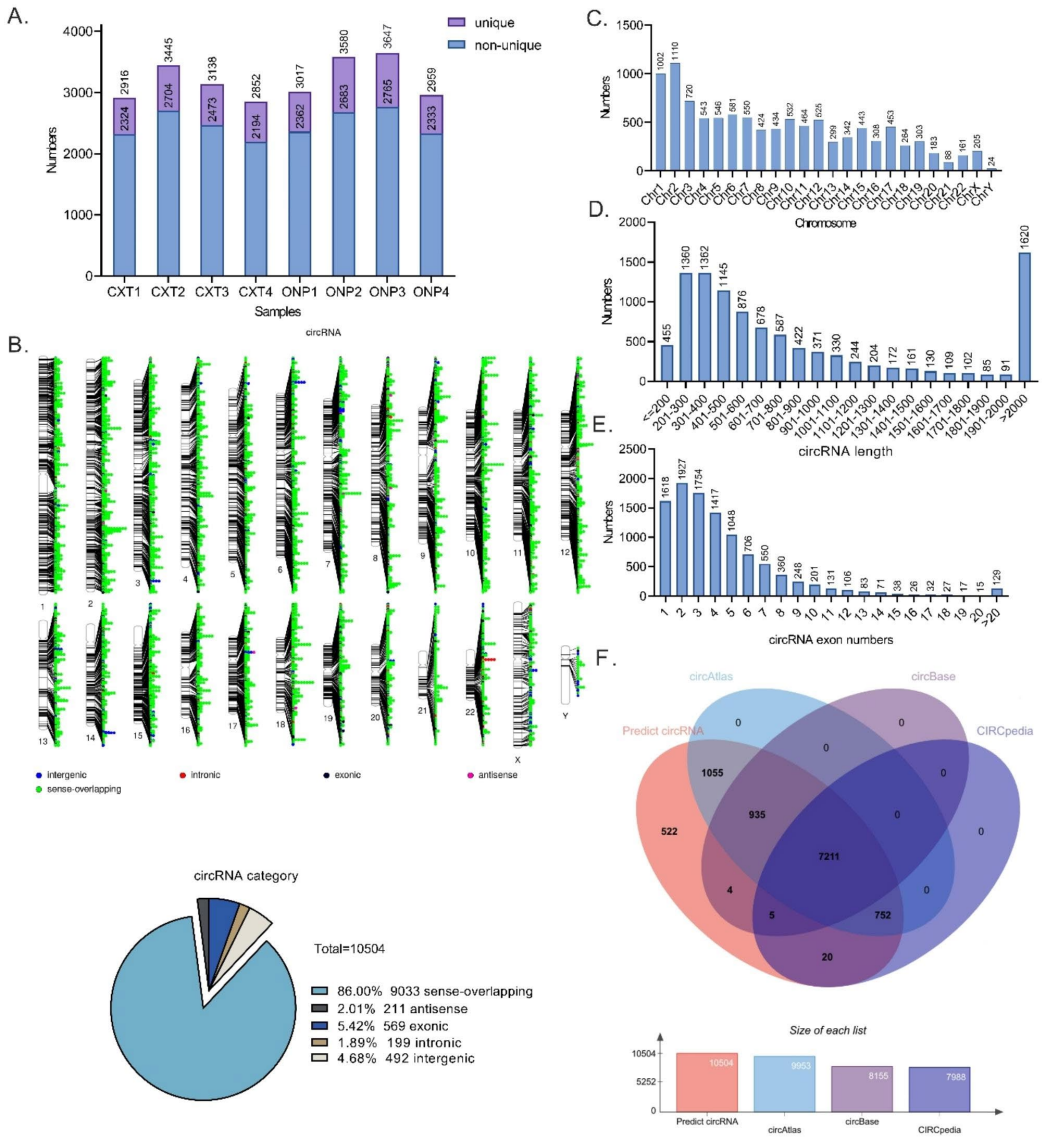
**Identification of novel circular RNAs (circRNAs) in oculomotor nerve palsy (ONP) and constant exotropia (CXT) samples.** (**A**) The number of circRNAs predicted in each medial rectus muscle sample. Unique circRNA numbers referred to the number of circRNAs predicted specifically in each sample compared to other samples. (**B**) CircRNA category and chromosome distribution. (**C**) Chromosome distribution of novel circRNAs. (**D**) Length distribution of novel circRNAs. (**E**) Exon numbers of novel circRNAs. (F) CircRNA comparison and annotation with databases

A total of 10,504 circRNAs were predicted from RNA sequencing data from the medial rectus muscle tissue samples of four ONP patients and four CXT patients. Among all the samples, ONP3 contained a relatively higher number of circRNAs (34.7%, 3,647/10,504), either for unique ones (882/10,504) or non-unique ones (2,765/10,504) (Fig. 1A). Classification of the positional relationship of circRNAs to known protein-coding transcripts showed that 86.0% (9,033/10,504) of novel circRNAs belonged to sense-overlapping regions, which comprised the largest category (Fig. 1B).

The chromosome and length distribution results showed that novel circRNAs were mainly enriched on Chr2 (10.6%, 1,110/10,504) (Fig. 1C), and circRNAs with a length of more than 2,000 nt accounted for the largest proportion (15.4%, 1,620/10,504) (Fig. 1D). In addition, exon numbers of novel circRNAs were calculated, and circRNAs with two exons (18.3%, 1,927/10,504) comprised the largest category (Fig. 1E).

Moreover, we compared and annotated the predicted circRNAs with known circRNAs based on three databases, namely circAtlas, circBase and CIRCpedia v2. As a result, 68.7% (7,211/10,504) of the predicted circRNAs were included in all three databases (Fig. 1F).

### Expression profiles of differentially expressed circRNAs

**Fig. 2:**
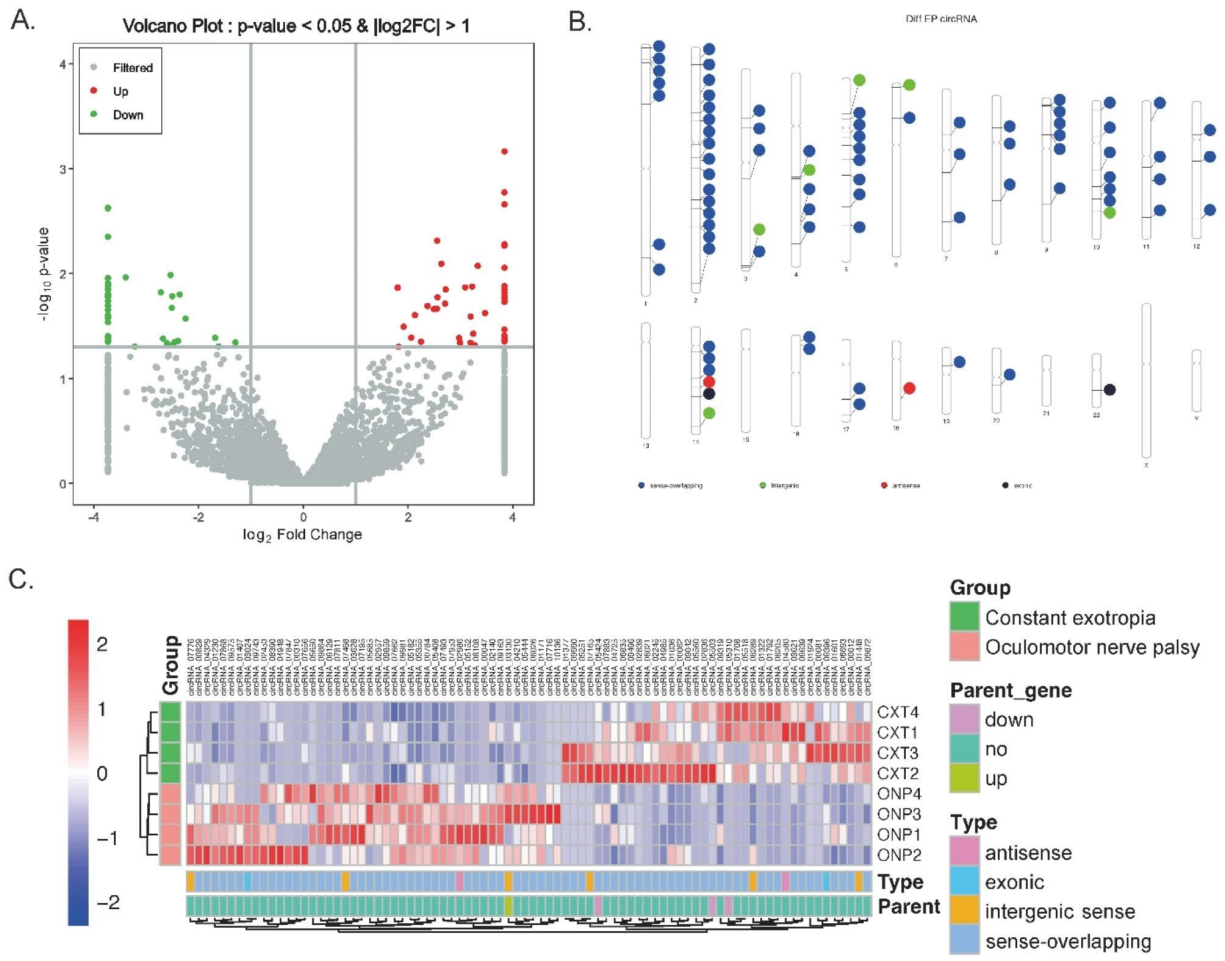
**Differentially expressed circRNAs between ONP and CXT groups.** (**A**) Volcano plot displayed differential circRNA expression between ONP and CXT patients. (**B**) Category and chromosome distribution of the differentially expressed circRNAs. (**C**) Hierarchical clustering heatmap demonstrated differential circRNA expression between medial rectus muscle samples of ONP and CXT groups, as well as the category and parent gene expression of the significantly differentially expressed circRNAs

A total of 84 circRNAs were identified as differentially expressed circRNAs with the cut-off thresholds of *P* value < 0.05 and |log2 FC| >1, including 46 up-regulated and 38 down-regulated circRNAs (Fig. 2A). These circRNAs were distributed across all regions of the genome except for Chr13, Chr15, Chr21, ChrX and ChrY, and were mainly located on sense-overlapping regions on Chr2 (Fig. 2B).

As revealed by a hierarchical clustering heatmap, the expression patterns of these differentially expressed circRNAs were notably different between the ONP and CXT groups. Moreover, the expression levels of the majority of the parent genes of the significantly differentially expressed circRNAs exhibited no significant change in the ONP samples (Fig. 2C).

### Functional enrichment analysis of the parent genes of differentially expressed circRNAs

**Fig. 3:**
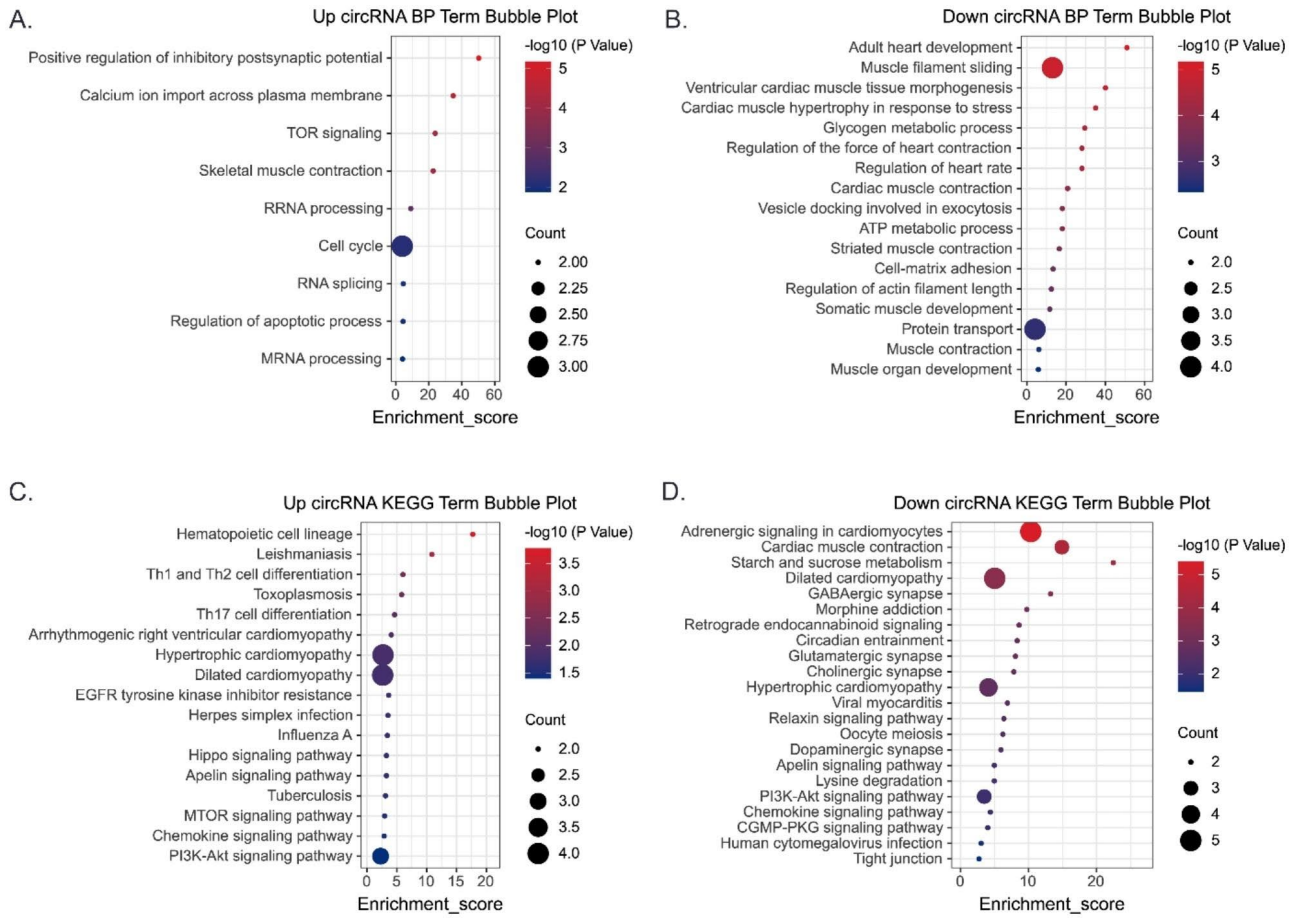
**Gene Ontology (GO) functional and Kyoto Encyclopedia of Genes and Genomes (KEGG) pathway enrichment analysis of the parent genes of differentially expressed circRNAs.** GO terms in the biological process (BP) category enriched for the parent genes of (**A**) up-regulated circRNAs and (**B**) down-regulated circRNAs. KEGG pathways enriched for the parent genes of (**C**) up-regulated circRNAs and (**D**) down-regulated circRNAs

GO and KEGG functional enrichment analyses were utilized among the parent genes of differentially expressed circRNAs to inspect the potential function of these circRNAs. GO analysis showed that the parent genes of up-regulated circRNAs were mainly enriched in calcium ion import across plasma membrane, TOR signaling, and skeletal muscle contraction in the BP category (Fig. 3A). Meanwhile, the parent genes of down-regulated circRNAs significantly correlated with muscle filament sliding, glycogen metabolic process and vesicle docking involved in exocytosis in the BP category (Fig. 3B).

KEGG pathway enrichment analysis printed that the parent genes of up-regulated circRNAs significantly correlated with hippo, apelin and mTOR signaling pathway (Fig. 3C). Meanwhile, the parent genes of down-regulated circRNAs mainly correlated with relaxin, apelin and PI3K-AKT signaling pathway (Fig. 3D).

### Construction of circRNA-miRNA-mRNA and circRNA-RBP interaction network

**Fig. 4:**
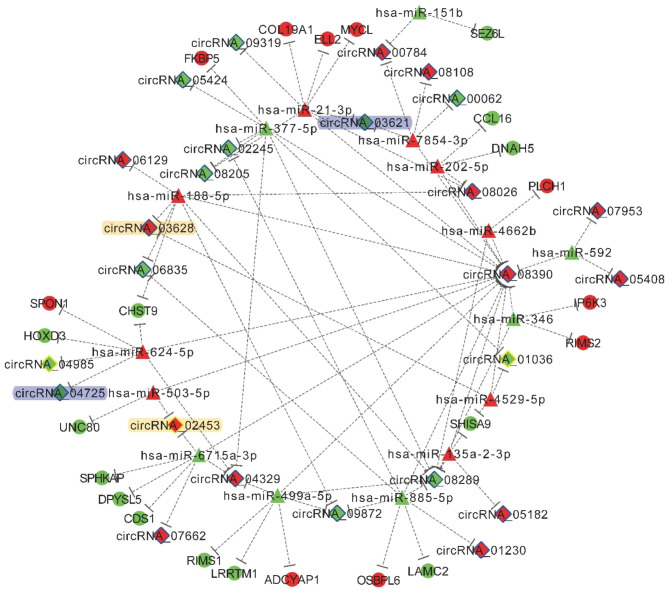
**CircRNA-microRNA (miRNA)-mRNA interaction network.** Diamonds, triangles and circles represent circRNAs, miRNAs and mRNAs, respectively. Red and green indicate up-regulation and down-regulation. The significantly up-regulated circRNAs were highlighted in yellow, and the significantly down-regulated circRNAs were highlighted in blue

In order to have a further insight into the roles of differentially expressed circRNAs in the pathological alterations of EOMs in ONP, a circRNA-miRNA-mRNA interaction network was constructed. As a result, up-regulated circRNA_03628 significantly interacted with hsa-miR-188-5p and hsa-miR-4529-5p, while up-regulated circRNA_02453 significantly correlated with hsa-miR-503-5p and hsa-miR-6715a-3p. In addition, down-regulated circRNA_03621 was associated with hsa-miR-21-3p and hsa-miR-7854-3p, while down-regulated circRNA_04725 correlated with hsa-miR-624-5p (Fig. 4).

**Fig. 5:**
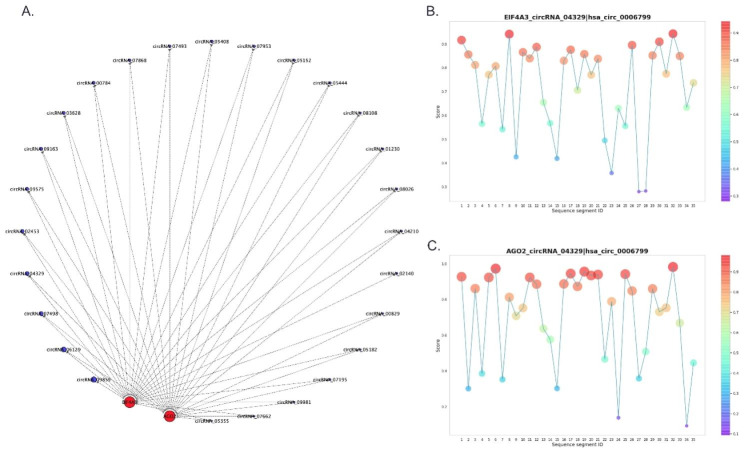
**Interaction between up-regulated circRNAs and RNA binding proteins (RBPs).** (**A**) Interaction network between AGO2, EIF4A3 and up-regulated circRNAs. Red and blue dots represent RBPs and circRNAs, respectively. Size of circles indicates the fold change of up-regulation. (**B**) Scores of interaction between EIF4A3 and each segment of circRNA_04329. (**C**) Scores of interaction between AGO2 and each segment of circRNA_04329

Moreover, RBPsuite was used to predict the potential proteins bound to up-regulated circRNAs. As a result, 37 proteins were screened out as RBPs for up-regulated circRNAs, among which Argonaute 2 (AGO2) and Eukaryotic initiation factor 4 A-3 (EIF4A3) were significantly interacted with 26 up-regulated circRNAs (< 4,000 nt), including circRNA_04329, circRNA_02453, circRNA_09575, circRNA_03628 and circRNA_02140 (Fig. 5A). As a representative up-regulated circRNA, circRNA_04329 was broken into 35 segments of 101 nucleotides, and the interactions between EIF4A3 (Fig. 5B), AGO2 (Fig. 5C) and each segment of circRNA_04329 were scored.

### RT-qPCR validation of differentially expressed circRNAs

**Fig. 6:**
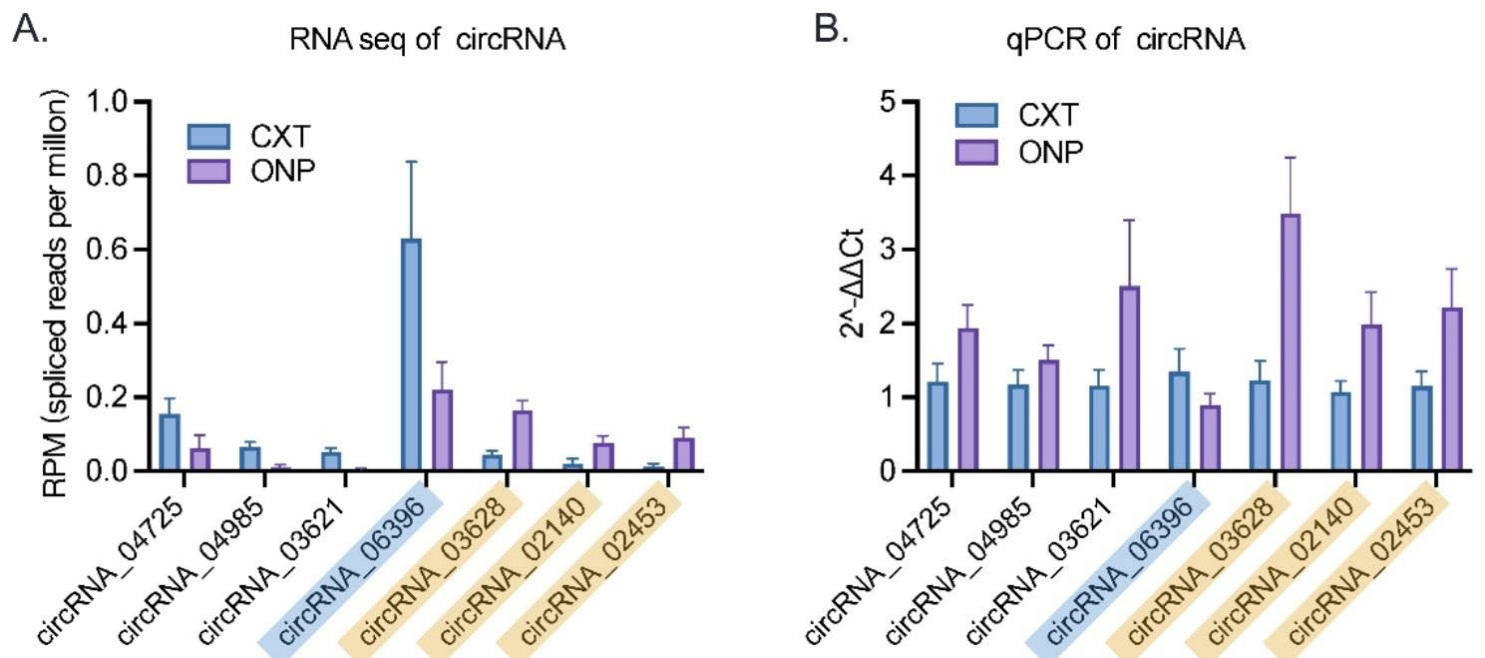
**Expression levels of differentially expressed circRNAs.** Expression levels of seven differentially expressed circRNAs (circRNA_04725, circRNA_04985, circRNA_03621, circRNA_06396, circRNA_03628, circRNA_02140 and circRNA_02453) according to (**A**) RNA sequencing data and (**B**) RT-qPCR results. The significantly up-regulated circRNAs were highlighted in yellow, and the significantly down-regulated circRNAs were highlighted in blue

The expression levels of seven differentially expressed circRNAs (circRNA_04725, circRNA_04985, circRNA_03621, circRNA_06396, circRNA_03628, circRNA_02140 and circRNA_02453) in medial rectus muscle samples of ONP and CXT patients were detected by RT-qPCR. Consistent with the RNA sequencing data, the expression levels of up-regulated circRNAs (circRNA_03628, circRNA_02140 and circRNA_02453) were increased in the ONP samples, while the down-regulated circRNA_06396 showed decreased expression in the ONP samples (Fig. 6). Notably, the up-regulation level of circRNA_03628 was the highest among all the up-regulated circRNAs, according to both RNA sequencing and RT-qPCR results. Therefore, circRNA_03628 was selected for further annotation and analysis.

### Verification, characterization and clinical significance of the up-regulated circRNA_03628

**Fig. 7:**
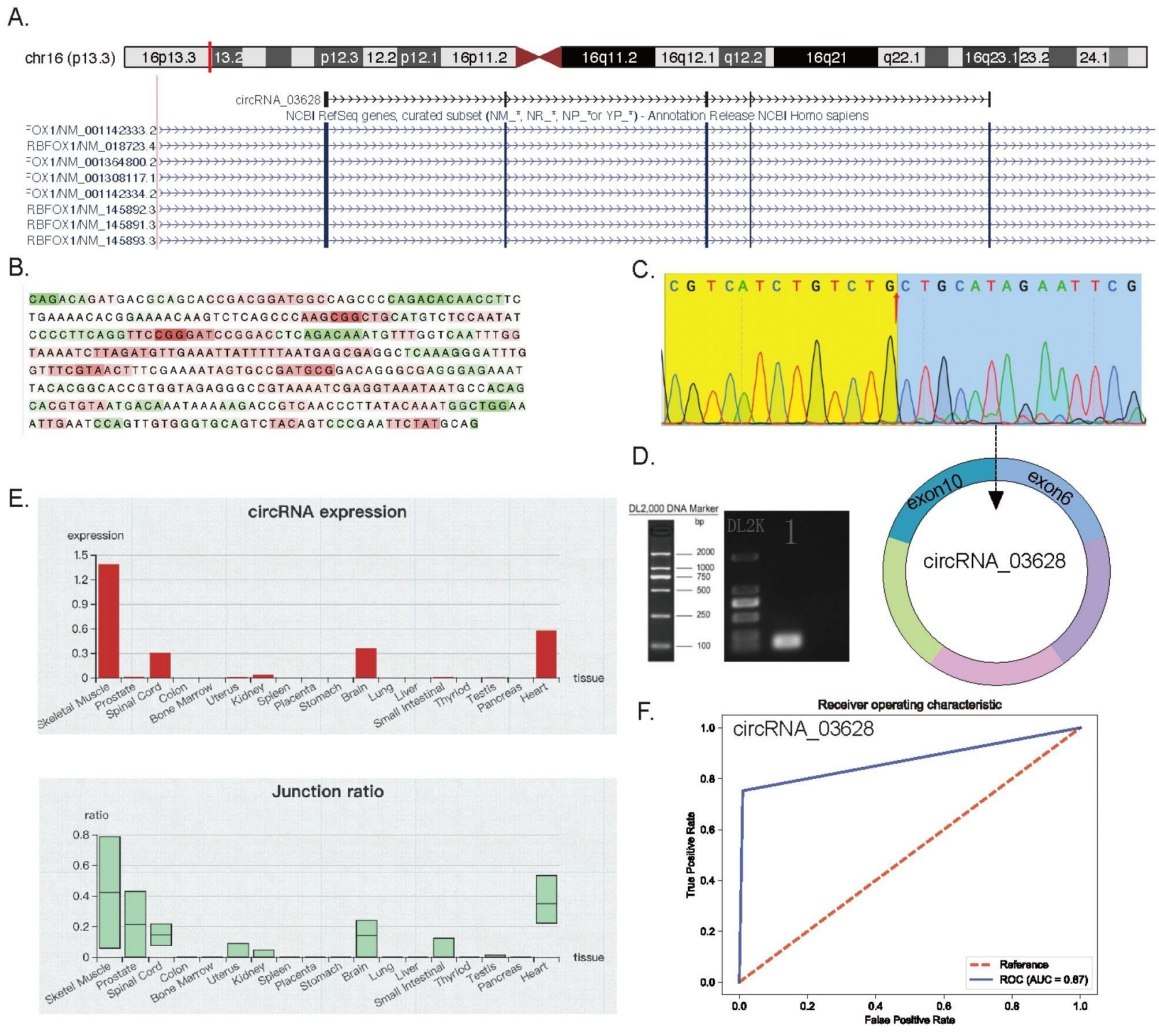
**Further insight into circRNA_03628.** (**A**) Localization of the parent gene of circRNA_03628 on chromosome 16. (**B**) Subcellular localization of circRNA_03628. Red indicates more cytoplasmic, whereas green indicates more nuclear. (**C**) Back-splice junction of circRNA_03628 was verified by Sanger sequencing. (**D**) PCR product of circRNA_03628 was verified by nucleic acid electrophoresis. (**E**) Expression of circRNA_03628 among multiple tissues. CircRNA_03628 was highly enriched in skeletal muscle tissue. (**F**) Receiver operating characteristic (ROC) curve of circRNA_03628

According to NCBI, circRNA_03628 was cyclized from exon 6 and 10 of the parent gene RBFOX1, which is localized on Chr16 (p13.3) (Fig. 7A). A deep learning model of lncLocator 2.0 using a stacked ensemble strategy indicated that circRNA_03628 was more likely to be located in cytoplasm rather than nucleus (Fig. 7B). Afterwards, the back-splice junction of circRNA_03628 was verified using Sanger sequencing of the PCR product of circRNA_03628 (Fig. 7C). Nucleic acid electrophoresis detected the product at 126 bp (Fig. 7D). The uncropped original electrophoresis gel can be seen in Supplementary Fig. 1. Furthermore, the expression of circRNA_03628 in multiple tissues was predicted by circAtlas 2.0. As a result, the expression level and junction ratio of circRNA_03628 were the highest in skeletal muscle tissue (Fig. 7E). ROC curve analysis was conducted to test the clinical significance of circRNA_03628. The AUC of circRNA_03628 was 0.87, suggesting its potential in the prediction and diagnosis of ONP (Fig. 7F).

## Discussion

Recently, a growing number of researchers have tried to illuminate the molecular features of EOMs in strabismus at both gene and protein levels. Altick et al. [42] found that the expression was predominantly up-regulated in genes involved in extracellular matrix (ECM) structure, and down-regulated in genes associated with muscle contractility in strabismic EOMs. In addition, protein and gene expressional quantification showed significant variations in strabismic EOMs with respect to important motor proteins, ECM factors and connective tissue [43]. These studies provided us a deeper insight into the gene expression profile of strabistic EOMs. However, the pathological characteristics of EOMs in ONP, especially the circRNA expression profile of EOMs in ONP remain unclear.

In this study, medial rectus muscle tissue samples from four ONP patients and four CXT patients were collected for high-throughput RNA sequencing. A total of 10,504 predicted circRNAs were identified, which were mainly distributed on Chr2 and located in sense-overlapping regions, with a length of more than 2,000 nt. Among the 84 differentially expressed circRNAs between the ONP and CXT groups, the expression levels of circRNA_03628, circRNA_02140 and circRNA_02453 were substantially increased in the ONP group, while the expression level of circRNA_06396 was significantly reduced, which was validated by RT-qPCR.

CircRNA_03628 was found to be highly expressed in SH-SY5Y cell lines [44], and was significantly down-regulated in human glioblastoma and oligodendroglioma compared to normal tissue [45]. CircRNA_02453 was first identified in Telomerized Hs68 human fibroblasts cells [46] and HEK293 cell lines [47], and was found to be down-regulated during SH-SY5Y differentiation [44]. A map of human circRNAs in clinical-relevant tissues revealed that circRNA_02453 is highly expressed in fibroblasts, muscle and right atrium [48]. CircRNA_02140 and circRNA_06396 were detected in Hela S-3 cell lines and their biogenesis were highly conserved and integral across species [49]. However, these studies all focused on the localization and expression levels of these circRNAs. The potential function of these circRNAs, especially their potential role on the pathophysiological processes of EOMs in ONP has not been reported.

Our study found that the parent genes of the up-regulated circRNAs were closely associated with skeletal muscle contraction, TOR signaling and hippo signaling pathway as revealed by functional enrichment analysis. TOR signaling pathway is a major monitor of cell growth and aging, which responds to nutrient availability, growth factor signals, and cellular stresses such as hypoxia and energy stress [50, 51]. According to Bodine et al. [52], the activation of Akt/mTOR pathway was associated with the regulation of skeletal muscle fiber size, and can prevent muscle atrophy caused by disuse. Hippo pathway is a complicated signaling network with over 30 components, which can avert adult tissue growth and modulate differentiation, proliferation and migration in cells and developing organs [53]. YAP/TAZ, the adaptor proteins of hippo pathway, are potent regulators of myogenesis and muscle size, and may suppress muscle atrophy [54].

In addition, the parent genes of down-regulated circRNAs significantly correlated with the contraction force of muscle fibers, relaxin signaling and apelin signaling. Relaxin is a hormone structurally associated with insulin and insulin-like growth factor, exerting its regulatory effect on the inflammation, tissue remodeling, and fibrosis of the skeletal muscle, mediated by different signaling pathways [55]. Apelin can promote muscle function by activating autophagy, myofiber mitochondriogenesis and anti-inflammatory pathways as well as strengthening the regenerative capacity by targeting muscle stem cells [56]. In summary, our findings suggested that the differentially expressed circRNAs revealed in the medial rectus muscle tissue samples of ONP patients might be involved in the pathological processes of EOMs in ONP.

In order to further elaborate the characteristics of differentially expressed circRNAs in the EOMs of ONP, circRNA-RBP interaction network was constructed. As a result, AGO2 and EIF4A3 significantly interacted with 26 up-regulated circRNAs, including circRNA_03628. AGO2 is the sole member with catalytic activity in the argonaute family that is of great significance during gene silencing processes guided by small RNAs, which also participates in gene regulation processes inside the nuclei, alternative polyadenylation and translational activation [57, 58]. EIF4A3 is a DEAD-box protein located in the nuclear matrix, which plays a vital role in splicing, RNA trafficking and nonsense-mediated decay, and macro-autophagy/autophagy [59, 60]. These results suggested that the differentially expressed circRNAs might play a regulatory role in the pathological changes of EOMs in ONP.

Among all the differentially expressed circRNAs, circRNA_03628 was up-regulated among all the samples in the ONP group based on the RNA sequencing data. Thus, we focused on circRNA_03628 for further elaboration. According to our analysis, circRNA_03628 might be a cytoplasmic circRNA cyclized from exon 6 and 10 of RBFOX1, which was highly enriched in skeletal muscle tissue. Besides, ROC curve analysis showed the diagnostic value of circRNA_03628 in ONP.

Furthermore, circRNA-miRNA-mRNA interaction network indicated that up-regulated circRNA_03628 was significantly related to hsa-miR-188-5p and hsa-miR-4529-5p. According to previous studies, hsa-miR-188-5p was up-regulated in breast cancer tissue [61] and the serum of dengue-infected patients [62] compared to healthy controls. Besides, the regulatory role of hsa-miR-188-5p has been found in tumor cell migration [63] and atrial fibrillation [64]. In addition, hsa-miR-4529-5p was found to be involved in the progression of gastric cancer [65]. Taken together, the results suggested that circRNA_03628 as well as its interaction with hsa-miR-188-5p and hsa-miR-4529-5p might play a crucial role in the pathological changes of EOMs in ONP.

Currently, there is no reliable biomarker for the prediction and diagnosis of ONP. Our findings suggested that circRNA_03628 might be a promising candidate of biomarkers for the prediction and diagnosis of ONP. However, a larger number of EOM samples from ONP patients, and peripheral blood samples if any, are needed to verify our findings, which is the main limitation of our study.

## Conclusions

To sum up, our study identified 84 differentially expressed circRNAs from the medial rectus muscle tissue samples between the ONP and CXT groups. Based on RNA sequencing data, the expression levels of circRNA_03628, circRNA_02140 and circRNA_02453 were substantially increased, while the expression level of circRNA_06396 was significantly reduced, as further validated by RT-qPCR. Functional enrichment analysis suggested that the differentially expressed circRNAs were closely related to skeletal muscle contraction. In addition, circRNA-RBP interaction analysis found that AGO2 and EIF4A3 were significantly interacted with 26 up-regulated circRNAs including circRNA_03628. The localization, characteristics and clinical significance of circRNA_03628 were further revealed, as well as its interaction with has-miR-188-5p and hsa-miR-4529-5p, suggesting its potential role in the pathogenesis and clinical evaluation of ONP. These findings may provide novel insights into the molecular biology of EOMs in ONP.

### Electronic supplementary material

Below is the link to the electronic supplementary material.

Supplementary Material 1

Supplementary Material 2

## Data Availability

The sequencing data in this study have been uploaded to online repositories. The name of the repository and accession number can be found below: https://www.ncbi.nlm.nih.gov/bioproject/PRJNA903490/, PRJNA903490.
