# Supplementary material for: Identification and characterization of differentially expressed circular RNAs in extraocular muscle of oculomotor nerve palsy
Source: BMC Genomics. 2023 Oct 17;24:617. doi: 10.1186/s12864-023-09733-3 (PMC10583365; doi:10.1186/s12864-023-09733-3)
Supplement: Supplementary file 1 — Supplementary Material 1 [file 12864_2023_9733_MOESM1_ESM.pdf]

Supplementary Table 1. Quality control details for total and small RNA sequencing.

Quality control for total RNA sequencing

Sample	RawReads	CleanReads	ValidBases	Total mapped
**CXT1**	101.60M	98.16M	89.96%	97.77%
**CXT2**	113.75M	109.79M	89.64%	97.47%
**CXT3**	112.19M	108.66M	91.02%	98.05%
**CXT4**	116.57M	110.78M	88.29%	97.76%
**ONP1**	101.39M	98.44M	89.81%	97.75%
**ONP2**	105.24M	101.89M	89.59%	97.84%
**ONP3**	115.27M	111.61M	90.47%	97.83%
**ONP4**	117.43M	114.06M	90.66%	97.75%

Quality control for small RNA sequencing

Sample	RawReads	CleanReads	ValidBases	Aligned (%)
**CXT1**	26.32M	24.25M	92.14%	92.62
**CXT2**	26.3M	24.0M	91.25%	92.59
**CXT3**	26.49M	24.19M	91.32%	95.09
**CXT4**	26.58M	24.21M	91.08%	87.94
**ONP1**	26.06M	24.23M	92.98%	88.12
**ONP2**	26.87M	24.7M	91.92%	91.16
**ONP3**	27.27M	24.77M	90.83%	92.39
**ONP4**	26.04M	23.82M	91.47%	90.74
