# Supplementary material for: Identification and characterization of differentially expressed circular RNAs in extraocular muscle of oculomotor nerve palsy
Source: BMC Genomics. 2023 Oct 17;24:617. doi: 10.1186/s12864-023-09733-3 (PMC10583365; doi:10.1186/s12864-023-09733-3)
Supplement: Supplementary file 2 — Supplementary Material 2 [file 12864_2023_9733_MOESM2_ESM.pdf]

Supplementary Figure 1. Original gel of nucleic acid electrophoresis.

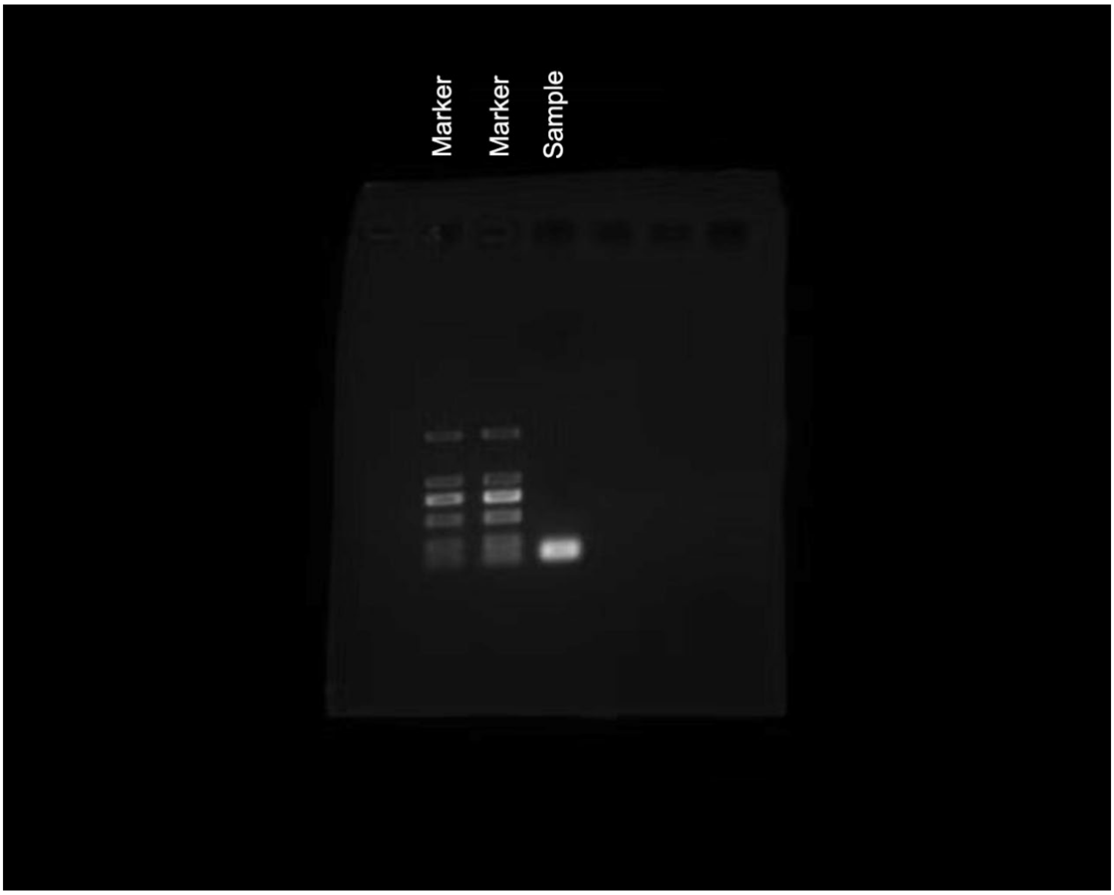

The PCR product of circRNA_03628 was detected using nucleic acid electrophoresis. Product length: 126 bp.
